# LncRNA SND1-IT1 facilitates TGF-β1-induced epithelial-to-mesenchymal transition via miR-124/COL4A1 axis in gastric cancer

**DOI:** 10.1038/s41420-021-00793-6

**Published:** 2022-02-19

**Authors:** Yang-Zhi Hu, Zhi-Li Hu, Tian-You Liao, Yuan Li, Yun-Long Pan

**Affiliations:** 1 Department of Gastrointestinal Surgery, Affiliated Hospital of Xiangnan University, Chenzhou, Hunan Province People’s Republic of China; 2 Department of Gastrointestinal Surgery, Liuzhou People’s Hospital, Liuzhou, Guangxi Province People’s Republic of China; 3 Department of Gastrointestinal Surgery, Shunde Hospital, Southern Medical University (The First People’s Hospital of Shunde Foshan), Foshan, Guangdong Province People’s Republic of China; 4 Department of Operation Room, Affiliated Hospital of Xiangnan University, Chenzhou, Hunan Province People’s Republic of China; 5 Department of General Surgery, The First Affiliated Hospital of Jinan University, Guangzhou, Guangdong Province People’s Republic of China

**Keywords:** Cancer, Diseases

## Abstract

The transformation of tumor cells from an epithelial to a mesenchymal-like phenotype, designated as epithelial-to-mesenchymal transition (EMT), represents a key hallmark of human cancer metastasis, including gastric cancer (GC). However, a large set of non-coding RNAs have been studied for their functions that initiate or inhibit this phenotypic switch in GC cells by regulating oncogenes or tumor suppressors. In this paper, we aimed to identify lncRNA SND1-IT1, miR-124, and COL4A1 gene in the context of GC with a specific focus on their effects on transforming growth factor β1 (TGF-β1)-induced EMT. The study included 52 paired samples of lesion tissues and adjacent lesion-free tissues surgically resected from patients diagnosed with GC. HGC-27 cells were stimulated with exogenous TGF-β1 (2 ng/mL). Expression of lncRNA SND1-IT1, miR-124, and COL4A1 was determined by RT-qPCR. CCK-8 assays, Transwell assays, immunoblotting analysis of EMT-specific markers, and tumor invasion markers were performed to evaluate cell viability, migration, and invasion of cultured HGC-27 cells. Luciferase activity assay was employed to examine miR-124 binding with lncRNA SND1-IT1 and COL4A1, respectively. LncRNA SND1-IT1 was upregulated in GC tissues and cells. TGF-β1-stimulated EMT and regulated lncRNA SND1-IT1, miR-124, and COL4A1 expressions in HGC-27 cells. LncRNA SND1-IT1 knockdown tempered HGC-27 cell viability, migration and invasion. LncRNA SND1-IT1 participated in TGF-β1-stimulated EMT in GC by sponging miR-124. MiR-124 attenuated TGF-β1-stimulated EMT in GC by targeting COL4A1. These results primarily demonstrated TGF-β1 can regulate cancer cell migration, invasion and stimulate EMT through the SND1-IT1/miR-124/COL4A1 axis in GC.

## Introduction

Gastric cancer (GC) is one of the most lethal malignancies and contributes to a substantial health care burden to patients. Each year, more than 1 million new cases have been diagnosed worldwide [1]. Although the past decade has witnessed a better understanding in prevention and personalized treatment, GC still remains the sixth in incidence and second in mortality globally in 2018 [2]. GC is a complex, heterogeneous disease arising from unique epidemiologic backgrounds, such as *Helicobacter pylori* infection [3], diet, smoking, obesity [4], host genetic mutation and instability [5], such as E-cadherin gene (CDH1), PALB2, BRCA1, and RAD51C mutations [6, 7]. Laparoscopy-assisted distal gastrectomy (LADG) and open distal gastrectomy (ODG) have been suggested as standard treatment options for GC at an early stage [8]. Due to the lack of marked symptoms in an early stage of GC, many patients present advanced disease at their initial diagnosis [9]. Epithelial–mesenchymal transition (EMT) is a prominent process in tumor development by converting epithelial phenotypic cells into mesenchymal phenotypic cells [10]. Transforming growth factor (TGF)-β1 is a well-characterized contributor to the process of EMT [11]. EMT usually limits total surgical resection and leads to therapeutic resistance, whereas there is limited knowledge about signaling pathways and effector molecules that initiate this phenotypic switch in GC. Thus, molecular processes and the downstream mechanisms involved in TGF-β1-stimulated EMT in GC remain enigmatic and merit further investigation.

Long non-coding RNAs (lncRNAs), a subgroup of RNAs with a length of more than 200 nucleotides, are abnormally expressed in various human cancers including GC [12]. Staphylococcal nuclease and Tudor domain-containing 1 intronic transcript 1 (SND1-IT1) is a newly identified lncRNA located on chromosome 7 (7q32.1) and found to modulate the promoter of oncogenes [13]. LncRNA SND1-IT1 has been reported to participate in the development of human diseases, including myocardial ischemia/reperfusion injury [14] and laryngeal squamous cell carcinoma [15]. However, the regulating signaling of lncRNA SND1-IT1 in GC development remains unknown. Plentiful lncRNAs govern a broad spectrum of biological processes, such as tumor initiation and metastasis by functioning as microRNA (miRNA) sponges and positive regulators of parental gene transcription [16]. The competitive endogenous RNA (ceRNA) hypothesis proposes that transcripts, including lncRNAs, share miRNA binding sites and compete for post-transcriptional control, which is becoming a new paradigm of ncRNA regulation [17]. For example, lncRNA SND1-IT1 has been suggested to promote osteosarcoma cancer cell growth by acting as a sponge to positively regulate POU2F1, the target of miR-655 (PMID: 31799644). An lncRNA-miRNA binding prediction indicates lncRNA SND1-IT1 and miR-124 share binding sites. MiR-124 was downregulated and characterized as a tumor suppressor in GC [18, 19]. There is a feedback loop between miR-124 and TGF-β pathway that is crucial for NSCLC metastasis [20]. Another miRNA-mRNA prediction suggests putative miR-124 binding sites on the 3′UTR of the COL4A1. Accordingly, mutations in COL4A1 are pleiotropic and tightly linked with a broad spectrum of human diseases, including GC [21, 22]. Herein, we proposed a hypothesis that lncRNA SND1-IT1 engages GC progression and metastasis by enhancing TGF-β1-stimulated EMT through modulating miR-124 and COL4A1.

The present study demonstrated that lncRNA SND1-IT1 was notably upregulated in GC tissues. Moreover, lncRNA SND1-IT1 could function as a ceRNA to regulate COL4A1 by competing for miR-124, which underpins TGF-β1-stimulated EMT in GC. These findings extend our knowledge of the biological role and underlying mechanism of lncRNA SND1-IT1 in GC metastasis.

## Results

### LncRNA SND1-IT1 was upregulated in GC tissues and cells

**Fig. 1:**
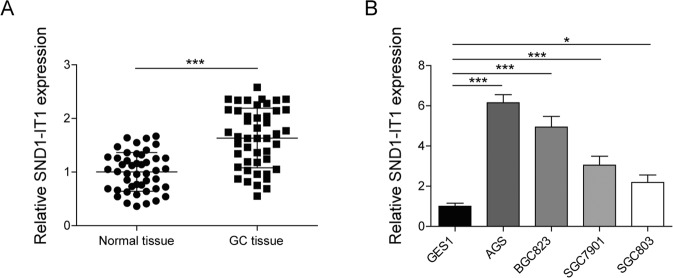
LncRNA SND1-IT1 is upregulated in GC tissues and cells. **A** The expression level of LncRNA SND1-IT1 was determined in tumor tissues (*n* = 52) by RT-qPCR and compared to adjacent non-tumor tissues (*n* = 52). **B** The expression level of lncRNA SND1-IT1 was determined in four selected GC cells by RT-qPCR and compared to GES1 cells. The results of **A** and **B** were normalized to the GAPDH expression. Values are expressed as mean ± SD from three technical repeats. **P < 0.01, ***P < 0.001.

Initially, lncRNA SND1-IT1 was quantified in human cancer tissue samples surgically resected from GC patients by RT-qPCR. As depicted in Fig. 1A, the results showed that the expression level of lncRNA SND1-IT1 was higher in lesion tissues than in lesion-free tissues. It revealed that relative expression of lncRNA SND1-IT1 was associated with invasion depth, lymph node metastasis, and TNM stage (*P* < 0.05, Table 1). We also found that lncRNA SND1-IT1 exhibited a higher expression level in four selected GC cells (HGC-27, AGS, NCI-N87, and MKN74) than in GES1 cells (Fig. 1B). Thus lncRNA SND1-IT1 was overexpressed in the context of GC.

**Table 1:** Relative expression levels of SND1-IT1 according to clinicopathological parameters.

Parameter	SND1-IT1 expression		*P*-value
	High expression	Low expression	
Age			
<45	17	22	0.4180
≥45	35	30	
Gender			
Female	29	33	0.5491
Male	23	19	
Tumor size			
<5 cm	16	24	0.1579
≥5 cm	34	28	
Invasion depth			
T2–T4	30	15	0.0053
Tis and T1	22	37	
Lymph node metastasis			
Negative	29	17	0.0294
Positive	23	35	
TNM stage			
I + II	16	31	0.0055
III + IV	36	21	

### LncRNA SND1-IT1 knockdown inhibited GC cell proliferation, migration, invasion, and EMT

**Fig. 2:**
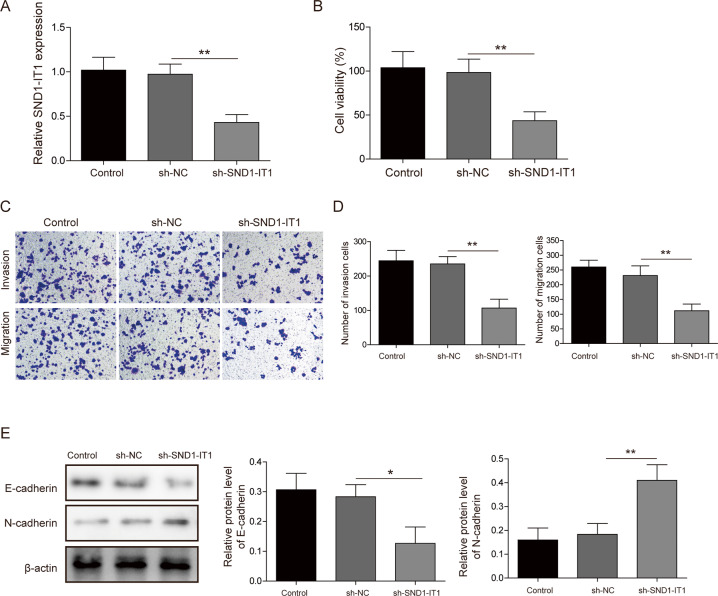
LncRNA SND1-IT1 knockdown inhibited GC cell proliferation and TGF-β1-induced EMT. **A** sh-SND1-IT1 was constructed in HGC-27 cells. Subsequent analyses were performed in untreated HGC-27 cells, HGC-27 cells expressing empty vector, and HGC-27 cells expressing lncRNA SND1-IT1. **B** HGC-27 cell viability was examined by CCK-8 assays. **C** Representative photomicrographs of HGC-27 cells migrating or invading from upper transwell chambers into bottom ones. **D** Quantitative analysis of HGC-27 cells migrating or invading from upper transwell chambers into bottom ones. **E** Representative immunoblots and quantitative analysis of E-cadherin, N-cadherin in HGC-27 cells. The results were normalized to the β-actin expression. Values are expressed as mean ± SD from three technical repeats. **P < 0.01.

HGC-27 cells were transfected with siRNA targeting lncRNA SND1-IT1 and then cultured without the addition of TGF-β1. The siRNA targeting lncRNA SND1-IT1 significantly knocked down lncRNA SND1-IT1 in HGC-27 cells. As shown in Fig. 2A, si-SND1-IT1 HGC-27 cells were constructed. The results showed that knockdown of lncRNA SND1-IT1 inhibited HGC-27 cell viability, migration, and invasion (Fig. 2B–D). To better understand whether lncRNA SND1-IT1 functionally influences migration and invasion properties of GC cells, we performed immunoblotting to determine alternations of (Fig. 2E), Not surprisingly, the results of immunoblotting showed a declined endogenous E-cadherin expression yet increased endogenous N-cadherin expression in si-SND1-IT1 HGC-27 cells. These results suggested that knockdown lncRNA SND1-IT1 led to inhibited GC cell migration and invasion along with enhanced EMT.

### TGF-β1-stimulated EMT and regulated lncRNA SND1-IT1, miR-124, and COL4A1 expressions in GC

**Fig. 3:**
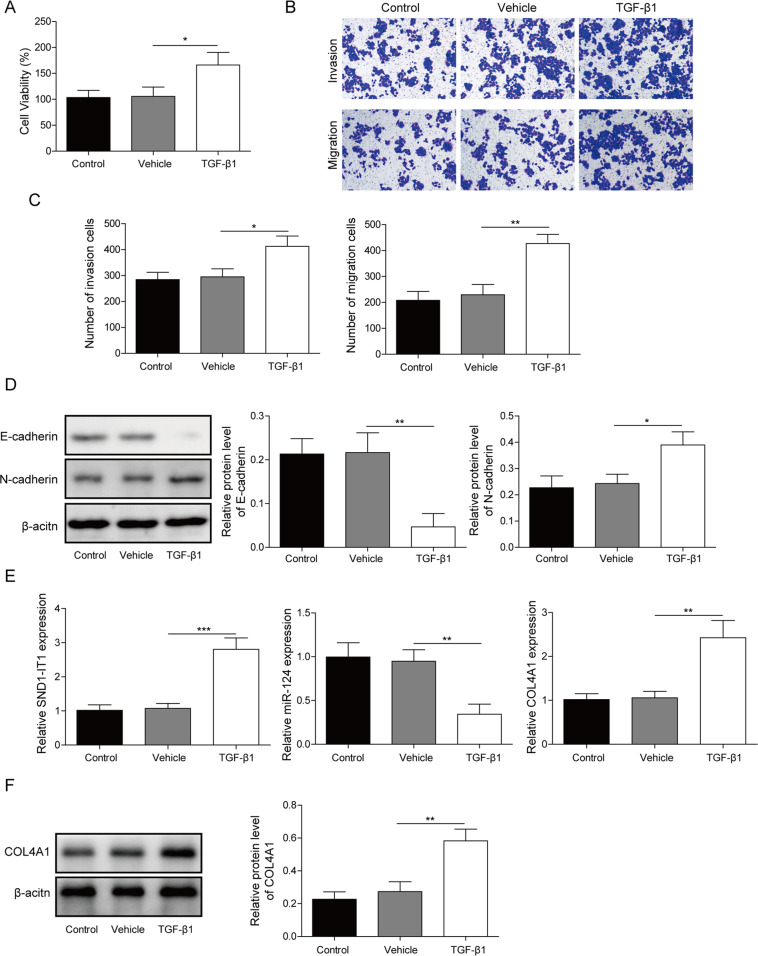
TGF-β1-stimulated EMT and regulated lncRNA SND1-IT1, miR-124, and COL4A1 expressions. **A** CCK-8 assays were performed to examine HGC-27 cell viability after TGF-β1 stimulation. **B** Representative photomicrographs of HGC-27 cells migrating or invading from upper transwell chambers into bottom ones after TGF-β1 stimulation. **C** Quantitative analysis of HGC-27 cells migrating or invading from upper transwell chambers into bottom ones after TGF-β1 stimulation. **D** Representative immunoblots and quantitative analysis of E-cadherin, N-cadherin in HGC-27 cells with TGF-β1 stimulation. The results were normalized to the β-actin expression. **E** The expression levels of lncRNA SND1-IT1, miR-124, and COL4A1 were determined in HGC-27 cells with TGF-β1 stimulation by RT-qPCR. **F** Representative immunoblots and quantitative analysis of COL4A1 in HGC-27 cells with TGF-β1 stimulation. Values are expressed as mean ± SD from three technical repeats. *P < 0.05, **P < 0.01.

TGF-β1 can enhance tumor metastasis by stimulating the process of EMT in GC [23]. In this part, HGC-27 cells were cultured with or without the addition of TGF-β1. The results obtained from CCK-8 and transwell assays displayed TGF-β1 stimulation facilitated HGC-27 cell viability, migration, and invasion (Fig. 3A–C). As we expected, the immunoblotting demonstrated TGF-β1 stimulation resulted in a declined E-cadherin with an elevated N-cadherin (Fig. 3D). Results of qRT-PCR revealed that TGF-β1 stimulation led to elevated expressions of lncRNA SND1-IT1 and COL4A1, as well as reduced miR-124 expression (Fig. 3E). As we expected, the immunoblotting demonstrated TGF-β1 stimulation resulted in an elevated COL4A1 (Fig. 3F). The aforementioned results showed that TGF-β1-stimulated EMT and regulated lncRNA SND1-IT1, miR-124, and COL4A1 expressions.

### LncRNA SND1-IT1 participated in TGF-β1-stimulated EMT in GC

**Fig. 4:**
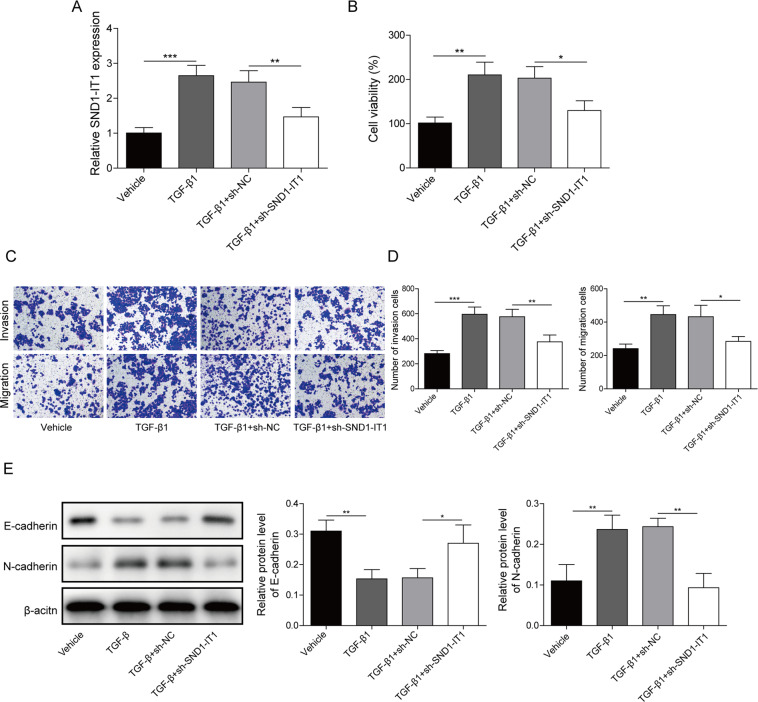
LncRNA SND1-IT1 participated in TGF-β1-stimulated EMT in GC. **A** LncRNA SND1-IT1 knockdown HGC-27 cells were constructed by delivering shRNA targeting lncRNA SND1-IT1. Subsequent analyses were performed in TGF-β1-treated HGC-27 cells with or without lncRNA SND1-IT1 knockdown. **B** CCK-8 assays were performed to examine HGC-27 cell viability. **C** Representative photomicrographs of HGC-27 cells migrating or invading from upper transwell chambers into bottom ones. **D** Quantitative analysis of HGC-27 cells migrating or invading from upper transwell chambers into bottom ones. **E** Representative immunoblots and quantitative analysis of E-cadherin and N-cadherin in HGC-27 cells. The results were normalized to the β-actin expression. Values are expressed as mean ± SD from three technical repeats. *P < 0.05, **P < 0.01.

HGC-27 cells were transfected with siRNA targeting lncRNA SND1-IT1 and then cultured with the addition of TGF-β1. The siRNA targeting lncRNA SND1-IT1 significantly knocked down lncRNA SND1-IT1 in HGC-27 cells (Fig. 4A). LncRNA SND1-IT1 knockdown was found to negate the effect of TGF-β1 stimulation and temper HGC-27 cell viability, migration, and invasion (Fig. 4B–D). As we expected, the immunoblotting also demonstrated lncRNA SND1-IT1 knockdown reversed the effect of TGF-β1 stimulation, reduced the N-cadherin, and increased the E-cadherin (Fig. 4E). Additionally, we found TGF-β1 stimulation negated the effect of lncRNA NEAT1 knockdown on these proteins. These data support the notion that TGF-β1 stimulates EMT by upregulating lncRNA SND1-IT1 expression in GC.

### LncRNA SND1-IT1 sponged miR-124 in GC

**Fig. 5:**
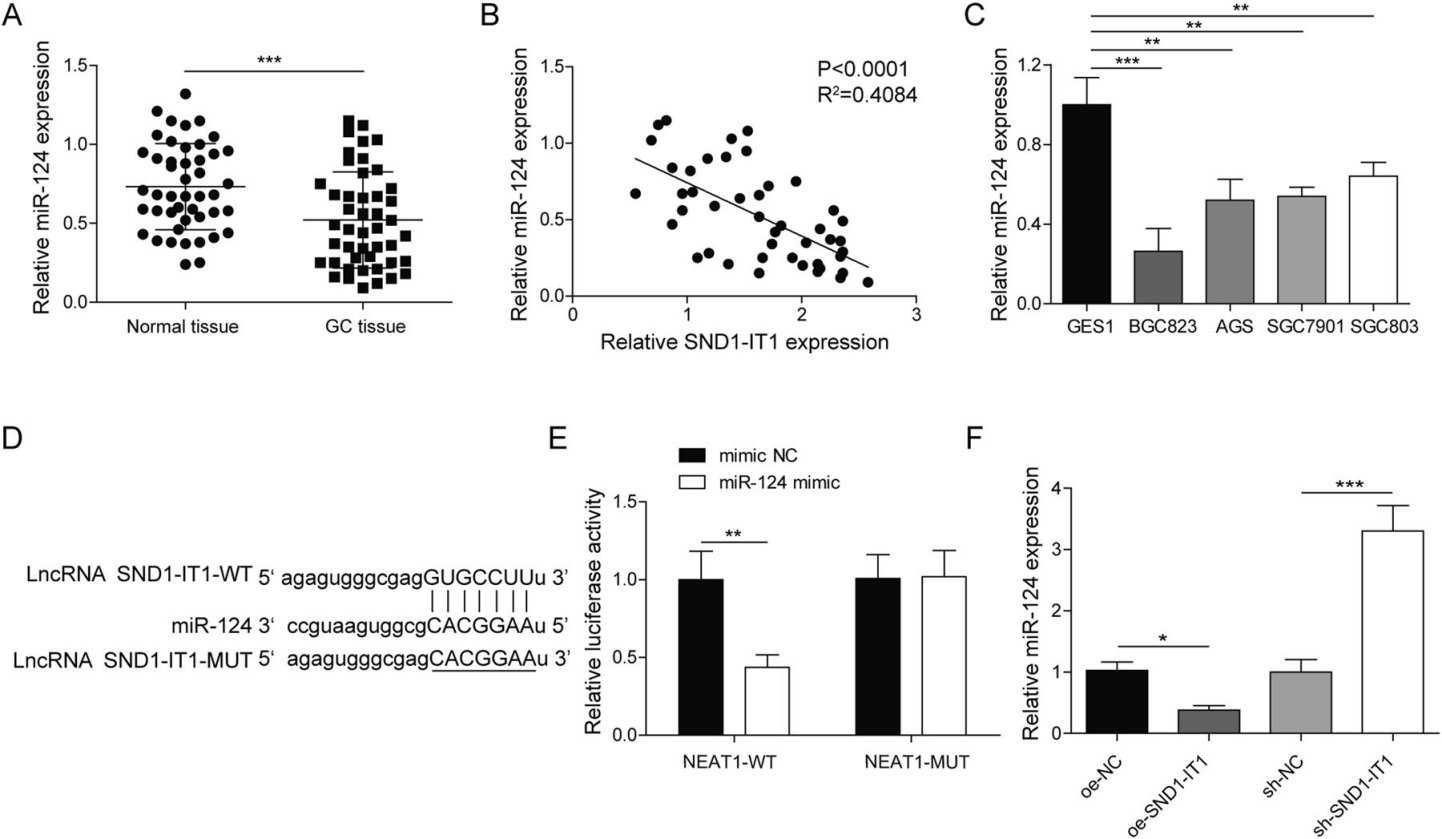
LncRNA SND1-IT1 sponged miR-124 in GC. **A** The expression level of miR-124 was determined in tumor tissues (*n* = 52) by RT-qPCR and compared to adjacent non-tumor tissues (*n* = 52). **B** Spearman correlation analysis between lncRNA SND1-IT1 and miR-124 in GC tissues. **C** The expression level of miR-124 was determined in four selected GC cells by RT-qPCR and compared to GES1 cells. **D** Putative miR-124 binding sites in the lncRNA SND1-IT1 transcripts by the starbase database (http://starbase.sysu.edu.cn/index.php). **E** Luciferase activity at the promoter of the reporter gene containing wt-lncRNA SND1-IT1 and mut-lncRNA SND1-IT1 was determined in the presence of miR-124 mimic. **F** MiR-124 expression was determined by RT-qPCR in HGC-27 cells upon lncRNA SND1-IT1 overexpression and knockdown, normalized to U6 expression. *P < 0.05, **P < 0.01, ***P < 0.001.

LncRNAs can act as competing endogenous RNAs (ceRNAs) to modulate gene expression by competing for the shared miRNAs in humans. Given that, we are curious about whether miR-124 is implicated in the regulation of lncRNA SND1-IT1 in the EMT in GC. We first quantified miR-124 expression in 52 paired samples of lesion tissues and lesion-free tissues surgically resected from GC patients by RT-qPCR. The results showed that miR-124 expression was lower in lesion tissues than in adjacent lesion-free tissues (Fig. 5A), and it was negatively correlated with lncRNA SND1-IT1 expression (Fig. 5B). Likewise, we examined miR-124 expression in four selected GC cells and GES1 and found that miR-124 exhibited a lower expression level in four selected GC cells than in GES1 cells (Fig. 5C). An lncRNA-miRNA prediction showed putative miR-124 binding sites in the lncRNA SND1-IT1 transcripts (Fig. 5D). LncRNA SND1-IT1 binding with miR-124 was further demonstrated by dual-luciferase reporter gene assay, evidenced by decreased luciferase luminosity at the promoter of the reporter gene containing wt-lncRNA SND1-IT1 compared with that containing mut-lncRNA SND1-IT1 in the presence of miR-124 mimic (Fig. 5E). Results of qRT-PCR indicated lncRNA SND1-IT1 overexpression reduced the miR-124, while lncRNA SND1-IT1 knockdown increased the miR-124 (Fig. 5F). These results indicated lncRNA SND1-IT1 sponged miR-124 in GC.

### LncRNA SND1-IT1 sponging miR-124 was involved in TGF-β1-stimulated EMT in GC

**Fig. 6:**
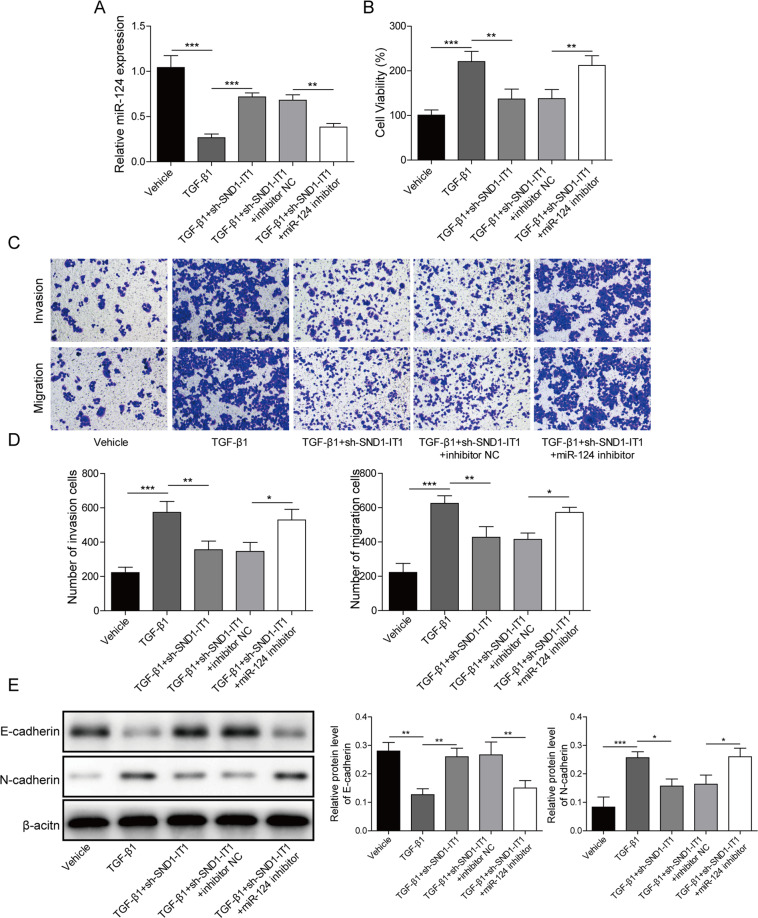
LncRNA SND1-IT1 sponging miR-124 was involved in TGF-β1-stimulated EMT in GC. **A** The expression level of miR-124 was determined by RT-qPCR in TGF-β1-stimulated HGC-27 cells with sh-SND1-IT1 and miR-124 inhibitor. **B** CCK-8 assays were performed to examine the viability in TGF-β1-stimulated HGC-27 cells with sh-SND1-IT1 and miR-124 inhibitor. **C** Representative photomicrographs of TGF-β1-stimulated HGC-27 cells migrating or invading from upper transwell chambers into bottom ones upon treatment with sh-SND1-IT1 and miR-124 inhibitor. **D** Quantitative analysis of TGF-β1-stimulated HGC-27 cells migrating or invading from upper transwell chambers into bottom ones upon treatment with sh-SND1-IT1 and miR-124 inhibitor. **E** Representative immunoblots and quantitative analysis of E-cadherin and N-cadherin in TGF-β1-stimulated HGC-27 cells treated with sh-SND1-IT1 and miR-124 inhibitor. The results were normalized to the β-actin expression. Values are expressed as mean ± SD from three technical repeats. *P < 0.05, **P < 0.01, ***P < 0.001.

We next performed a mechanistic investigation that lncRNA SND1-IT1 interaction with miR-124 governs TGF-β1-stimulated EMT in GC. We delivered shRNA targeting lncRNA SND1-IT1 and miR-124 inhibitor alone or together into HGC-27 cells in the presence of exogenous TGF-β1. As depicted in Fig. 6A, exogenous addition of TGF-β1 inhibited miR-124 expression, while following lncRNA SND1-IT1 knockdown further upregulate miR-124 expression in HGC-27 cells. CCK-8 and Transwell assays presented expected results that miR-124 inhibitor promoted HGC-27 cell viability, migration, and invasion, mimicked the effects of TGF-β1 stimulation, but reversed effects of lncRNA SND1-IT1 knockdown (Fig. 6B–D). Immunoblotting also showed a marked increase in E-cadherin concomitant with a decline in N-cadherin in TGF-β1-stimulated HGC-27 cells with lncRNA SND1-IT1 knockdown, whilst the results were reversed upon miR-124 inhibition (Fig. 6E). These data support the notion that lncRNA SND1-IT1 sponging miR-124 was involved in TGF-β1-stimulated EMT in GC.

### MiR-124 targeted and inhibited COL4A1 in GC

**Fig. 7:**
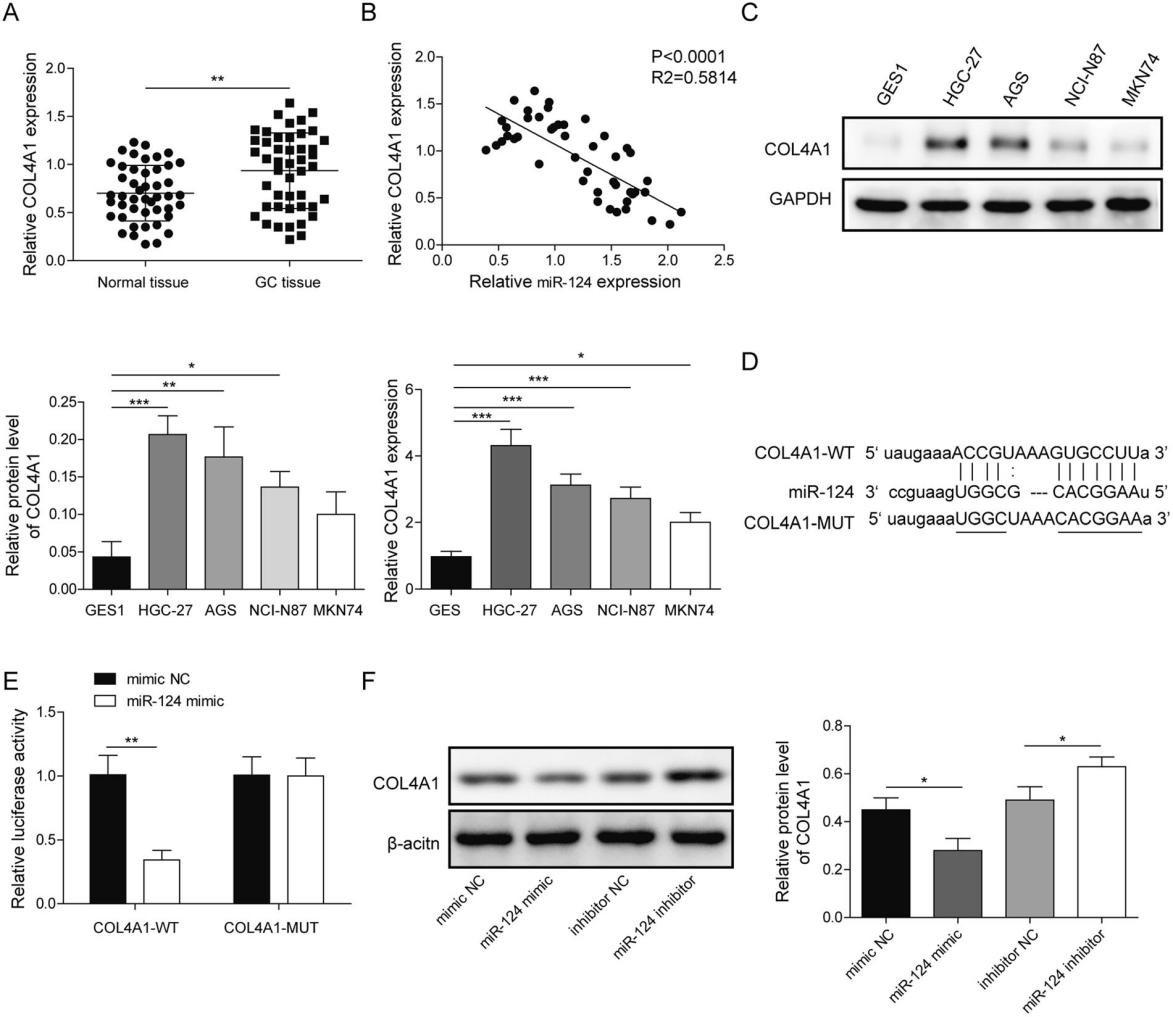
MiR-124 targeted and inhibited COL4A1. **A** The expression level of COL4A1 was determined in tumor tissues (*n* = 52) by RT-qPCR and compared to adjacent non-tumor tissues (*n* = 52). **B** Spearman correlation analysis between miR-124 and COL4A1 in GC tissues. **C** The expression level of COL4A1 was determined in four selected GC cells and GES1 cells by RT-qPCR and immunoblots. **D** Putative miR-124 binding sites in the COL4A1 mRNA 3′UTR in the starbase database. **E** Luciferase activity of the promoter of the reporter gene containing COL4A1-wt or COL4A1-mut in the presence of miR-124 mimic. **F** Representative immunoblots and quantitative analysis of COL4A1 protein in response to exogenous addition of miR-124 mimic and miR-124 inhibitor. Values are expressed as mean ± SD from three technical repeats. **P < 0.01, ***P < 0.001.

To the best of our knowledge, the miRNA regulatory network of a specific mRNA plays a crucial character in human diseases. We believed miR-124 regulating gene expression undertakes the regulation of lncRNA SND1-IT1 in GC. In the beginning, we examined COL4A1 expression in 52 paired samples of lesion tissues and lesion-free tissues by RT-qPCR. The results showed that COL4A1 expression was higher in lesion tissues than in lesion-free tissues (Fig. 7A), and it was negatively correlated with miR-124 expression (Fig. 7B). It was found that COL4A1 exhibited a higher expression level in four selected GC cells than in GES1 cells (Fig. 7C). We performed a web-available prediction of miR-124 binding sites in the COL4A1 mRNA 3′UTR in the starbase (Fig. 7D). We also observed a mark reduction in luciferase activity of the promoter of the reporter gene containing COL4A1-wt rather than COL4A1-mut in the presence of miR-124 mimic (Fig. 7E). In addition to dual-luciferase reporter gene assay, immunoblotting demonstrated targeted inhibition of COL4A1 by miR-124, as evidenced by declined COL4A1 in response to miR-124 mimic and increased COL4A1 in response to miR-124 inhibitor in HGC-27 cells (Fig. 7F). These data revealed that miR-124 targeted and inhibited COL4A1 in GC.

### MiR-124 attenuated TGF-β1-stimulated EMT in GC by targeting COL4A1

**Fig. 8:**
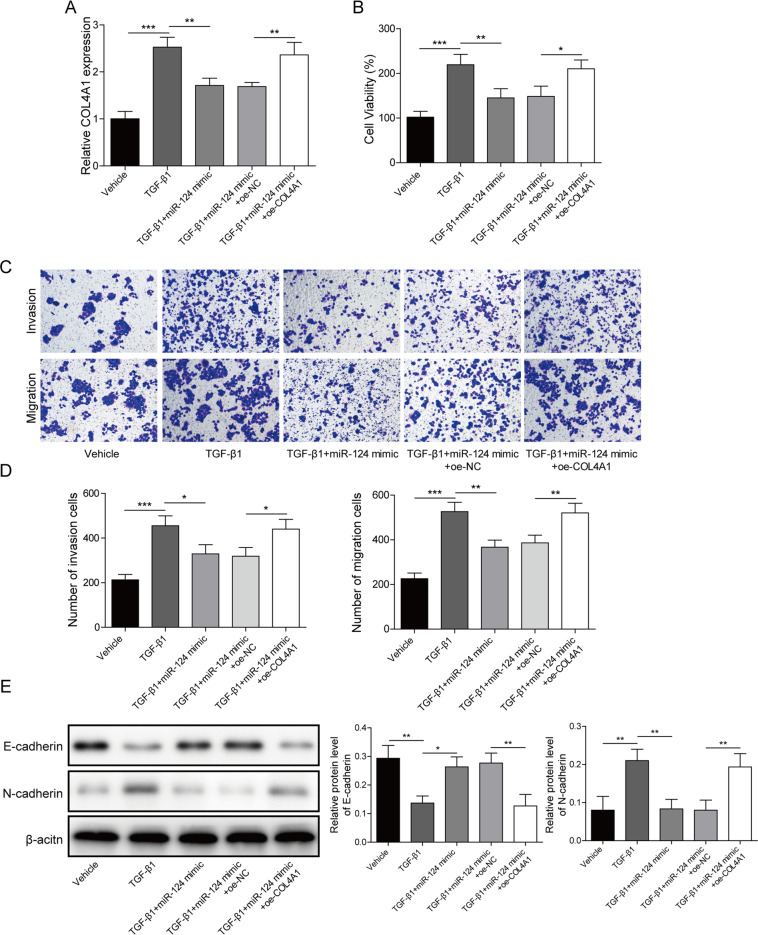
MiR-124 attenuated TGF-β1-stimulated EMT in GC by targeting COL4A1. **A** The expression level of COL4A1 was determined by RT-qPCR in TGF-β1-stimulated HGC-27 cells with miR-124 mimic and COL4A1 expression vector. **B** CCK-8 assays were performed to examine the viability in TGF-β1-stimulated HGC-27 cells with miR-124 mimic and COL4A1 expression vector. **C** Representative photomicrographs of TGF-β1-stimulated HGC-27 cells migrating or invading from upper transwell chambers into bottom ones upon treatment with miR-124 mimic and COL4A1 expression vector. **D** Quantitative analysis of TGF-β1-stimulated HGC-27 cells migrating or invading from upper transwell chambers into bottom ones upon treatment with miR-124 mimic and COL4A1 expression vector. **E** Representative immunoblots and quantitative analysis of E-cadherin and N-cadherin in TGF-β1-stimulated HGC-27 cells treated with miR-124 mimic and COL4A1 expression vector. The results were normalized to the β-actin expression. Values are expressed as mean ± SD from three technical repeats. *P < 0.05, **P < 0.01, ***P < 0.001.

Finally, we attempted to dissect out whether targeted inhibition of COL4A1 by miR-124 was involved in TGF-β1-stimulated EMT. The expression of COL4A1 was increased in HGC-27 cells after TGF-β1 treatment but this increase was prevented after miR-124 mimic treatment. In addition, it was observed the expression of COL4A1 was not different between HGC-27 cells with treatment of TGF-β1, miR-124 mimic, and COL4A1 expression vector and HGC-27 cells with TGF-β1 treatment alone (Fig. 8A). As shown in Fig. 8B–D, The contribution of TGF-β1 treatment to HGC-27 cell viability, migration, and invasion was negated by miR-124 mimic treatment. No significant difference concerning HGC-27 cell viability, migration, and invasion was observed between HGC-27 cells with treatment of TGF-β1, miR-124 mimic, and COL4A1 expression vector and HGC-27 cells with TGF-β1 treatment alone. Results of immunoblotting (Fig. 8E) displayed that a decrease in E-cadherin expression and an increase in N-cadherin expression following TGF-β1 treatment were reversed by miR-124 mimic treatment. No significant difference with regard to E-cadherin and N-cadherin expressions was found between HGC-27 cells with treatment of TGF-β1, miR-124 mimic, and COL4A1 expression vector and HGC-27 cells with TGF-β1 treatment alone. These results suggested that miR-124 attenuated TGF-β1-stimulated EMT in GC by targeting COL4A1.

## Discussion

Cancer metastasis is associated with EMT, which is a process whereby epithelial cells lose their polarity and acquire new features of mesenchyme. TGF-β1 has been reported as a predominant contributor in the process of EMT, but its mechanism remains to be elucidated [24]. Recently, a study showed that TGF-β1-induced upregulation of lncRNAs promoted GC invasion and migration [25]. In this study, we found TGF-β1 stimulation upregulated lncRNA SND1-IT1 and lncRNA SND1-IT1 promoted TGF-β1-stimulated EMT in GC. More than that, we also found lncRNA SND1-IT1 sponging miR-124 was involved in TGF-β1-stimulated EMT. Once miR-124 was inhibited, its target gene COL4A1 was triggered and thus enhanced TGF-β1-stimulated EMT. These results and our functional evidence for lncRNA SND1-IT1-miR-124-COL4A1 network might exhibit great value to explore the molecular mechanism underlying TGF-β1-stimulated EMT in GC.

LncRNA SND1-IT1 was found to be a promoter for metastasis of GC. As our results have shown, lncRNA SND1-IT1 was abundantly expressed in GC tissues and cells. Prior work has documented that lncRNA NEAT1 expression was enhanced in the context of osteosarcoma [26], suggesting its oncogenic role in human cancers. Additionally, cellular migration can be promoted by an accelerated EMT process in GC [27]. Not surprisingly, our results showed that overexpression of lncRNA SND1-IT1 led to a declined expression of E-cadherin but an increased N-cadherin expression in HGC-27 cells. The molecular pathogenesis of GC is closely associated with E-cadherin, and suppressed biological function of GC cells is correlated with upregulated E-cadherin level, considerably downregulated N-cadherin [28]. Moreover, we found TGF-β1 stimulation upregulated lncRNA SND1-IT1 and lncRNA SND1-IT1 promoted TGF-β1-stimulated EMT, as evidenced by an upregulated lncRNA SND1-IT1 in HGC-27 cells upon TGF-β1 stimulation. TGF-β1-induced upregulation of lncRNAs promoted GC invasion and migration [25]. TGF-β1 is known as a promoter of tumor metastasis by inducing EMT process in GC [29]. Following stimulation of TGF-β1, levels of Vimentin and α-SMA are increased while reduced E-cadherin expression is observed [30], which is in line with our data derived from this study. In contrast, knockdown of lncRNA SND1-IT1 suppressed EMT among GC cells by downregulating N-cadherin, Vimentin, and snail. The above-mentioned literature supports our finding that overexpression of lncRNA SND1-IT1 promoted cellular migration, invasion, and TGF-β1-stimulated EMT in GC cells.

The downstream molecular mechanism underlying lncRNA SND1-IT1 was subsequently explored. Our data exhibited that lncRNA SND1-IT1 could specifically bind to miR-124 which targeted and suppressed expression of COL4A1. Results derived from our study showed that miR-124 was downregulated in GC cells and tissues. Similarly, miR-124 has been found to be poorly expressed in GC [31]. Also, decreased expression of miR-124 has been associated with poor outcomes in GC patients [32], which demonstrated the tumor-suppressive role of miR-124 in GC. Moreover, upregulated miR-124 significantly suppresses cell proliferation, invasion, and downregulates levels of MMP2 and MMP9 among osteosarcoma cells [33]. We next found the regulatory mechanism between lncRNA SND1-IT1 and miR-124 in GC. Exogenous addition of TGF-β1 inhibited miR-124 expression, while following lncRNA SND1-IT1 knockdown further enhanced miR-124 expression in HGC-27 cells, suggesting the involvement of lncRNA SND1-IT1/miR-124/TGF-β1 in the process of EMT in GC. As previously reported, Zhang et al. demonstrated lncRNA UCA1/miR-124 axis modulates TGFβ1-induced EMT through JAG1/Notch signaling in tongue cancer [34]. COL4A1 directly targeted by miR-124 was found to be suppressed by miR-124. COL4A1 expression is reported to be associated with the TGFβ1 gene in subcutaneous adipose tissues [35]. Furthermore, another collagen gene family member, COL10A1 has been proved to induce EMT by increasing N-cadherin and Vimentin expression and reducing E-cadherin expression [36]. Our study further demonstrated that COL4A1 could negate the suppression of miR-124 elevation on TGFβ1-induced EMT. It could be indicated that overexpression of miR-124 restrained GC cell migration, invasion, and attenuated TGFβ1-induced EMT by silencing COL4A1 expression.

Conclusively, the study provides evidence that lncRNA SND1-IT1 could function as a ceRNA to regulate COL4A1 by competing for miR-124, which underpins TGF-β1-stimulated EMT in GC. However, further in vivo and in vitro investigations are required to validate whether there is positive feedback between lncRNA SND1-IT1 and TGFβ1, negative feedback between miR-124 and TGFβ1, and a ceRNA regulatory network of lncRNA SND1-IT1, miR-124, and COL4A1 in mediating the process of EMT in GC and all these regulatory mechanisms to explain TGFβ1-induced EMT in GC. Nevertheless, our data still revealed an oncogenic role for lncRNA SND1-IT1 in GC tumorigenesis, which may offer a strategy for using the lncRNA-based strategy as a potential therapeutic target for patients with GC.

## Materials and methods

### Human tissue specimens

The study included 52 paired samples of lesion tissues and lesion-free tissues from 52 GC patients who underwent gastrectomy at our hospital from February of 2018 to August of 2020. Among 52 patients, there were 42 males and 10 females, with age ranging from 32 to 68 years. No patient had received radiotherapy or chemotherapy before surgery. Sample collection was performed with informed consent for a protocol approved by the ethics committee of the hospital.

### Cell culture

We purchased four types of human GC cell lines AGS, HGC-27, NCI-N87, MKN74, and a human normal gastric epithelial cell GES1 from ATCC (USA), and all of them were harvested in the RPMI1640 (Gibco, Grand Island, NY, USA) with 10% fetal bovine serum (FBS, Hangzhou Sijichun, China), 100 U/L penicillin/100 mg/L streptomycin (Gibco). Cell harvest was performed in an incubator (37 °C, 5% CO_2_).

### Transient transfection

Two pcDNA3.1 constructs containing human SND1-IT1 full-length complementary DNA (cDNA) and COL4A1 cDNA, an anti-SND1-IT1 siRNA construct, miR-124 mimic, and miR-124 inhibitor and introduced into HGC-27 cells alone or in combination as required by using lipofectamine 2000 reagents in accordance with the instructions provided by the manufacturer (Invitrogen, USA). Forty-eight hours after transient transfection, HGC-27 cells were stimulated with exogenous TGF-β1 (2 ng/mL, Sigma Chemical, Aldrich Ltd.) for 24 h. All constructs, miR-124 mimic, miR-124 inhibitor, and their corresponding NC were synthesized by GenePharma (Shanghai, China).

### RNA extracts, reverse transcription, and real-time qPCR (RT-qPCR)

Total RNA was extracted using TRIzol reagents (Takara, Dalian, China). Synthesis of cDNA was carried out using the PrimeScript RT reagent Kit (RR047A, Takara, Dalian, China) following the instructions provided by the manufacturer. RT-qPCR was run on with the SYBR®Premix ExTaqTM II (Perfect Real Time) kit (DRR081, Takara, Japan) and analyzed using the PCR instrument (ABI, Foster City, CA, USA), with each reaction run in triplicate. Primers (Table 2) were generated by Sangon (Shanghai, China). MiR-124 expression level was normalized to U6 and SND1-IT1 expression level to GAPDH. Fold changes in expression were calculated by using 2^−^^ΔΔCt^. ΔCt represents the difference between the threshold cycle (Ct) for each target and housekeeping mRNA or miRNA levels.

**Table 2:** Primer sequences used for RT-qPCR.

Target	Sequences (5′–3′)
SND1-IT1-F	5′-CCTGAGCGGCAGATCAACC-3′
SND1-IT1-R	5′- AGGTAGATCATGCCATACTCTCG-3′
COL4A1-F	5′-CAGGTCCAAAGGGTGAAC-3′
COL4A1-R	5′-GTAGACCAACTCCAGGCT-3′
GAPDH-F	5′-GCACCGTCAAGGCTGAGAAC-3′
GAPDH-R	5′-TGGTGAAGACGCCAGTGGA-3′
miR-124-F	5′-GCCGCTAAGGCACGCG-3′
miR-124-R	5′-TATGGTTGTTCACGACTCCTTCAC-3′
U6-F	5′-ATTGGAACGATACAGAGAAGATT-3′
U6-R	5′-GGAACGCTTCACGAATTTG-3′

### Immunoblotting

Total protein was obtained in the RIPA buffer (Beyotime Biotechnology), and 50 μg of protein samples for each run were loaded into the wells of 10% SDS-PAGE for separation and then wet-transferred onto the poly (vinylidene fluoride) (PVDF) membrane. The membrane immunoreacted with antibodies (Abcam, Cambridge, UK) to E-cadherin (ab15148), N-cadherin (ab18203), Collagen type IV (ab6586), and β-actin (ab227387). Immunoblots were visualized by horseradish peroxidase-labeled IgG with the enhanced chemiluminescence (ECL) reagents (BB-3501, Amersham Pharmacia, UK) and quantified using a gel documentation system (Bio-Rad Quantity One Software v4.6.2, Bio-Rad Laboratories, Richmond, California, USA). The gray value of the targeted proteins was normalized to that of β-actin.

### CCK-8 assays

HGC-27 cells were seeded in 96-well plates (5 × 10^3^ per well) overnight. After treatment for 24 h, 10 μL CCK-8 reagents (Beyotime, Shanghai, China) were added to each well and further incubated for 2 h at 37 °C. Finally, the absorbance of HGC-27 cells was measured at 450 nm using a microplate reader (ThermoFisher Scientific).

### Transwell assays

Cell migration and invasion assays were performed in a 24-well transwell chamber (8-mm pore size). In brief, HGC-27 cells were subject to serum-free starvation for 24 h, and they were resuspended into 3 × 10^5^ cells/mL in serum-free DMEM. The suspension (100 μL) of HGC-27 cells were added into the upper chambers coated with (for cell invasion assays) or without (for cell migration assays) 50 μL Matrigel pre-incubated by a serum-free medium. Following 24-h incubation at 37 °C, the cells that transferred to the lower chamber containing 500 μL 10% FBS-supplemented DMEM were subject to 4% paraformaldehyde fixation (10 min) and 0.1% crystal violet staining (10 min). Stained cells were counted in six random microscopic fields.

### Dual-luciferase reporter gene assay

LncRNA SND1-IT1 transcripts or oligonucleotides on the COL4A1 mRNA 3′UTR containing the putative miR-124 binding sites (named lncRNA SND1-IT1-wt or COL4A1-wt) and lncRNA SND1-IT1 or COL4A1 mutated at the putative miR-124 binding sites (named lncRNA SND1-IT1-mut or COL4A1-mut) were artificially synthesized and inserted into a commercially available luciferase reporter vector pGL3 vector (Promega, Madison, WI, USA). Well-designed pMIR-reporter vectors with miR-124 mimic were delivered into HGC-27 cells (Shanghai Beinuo Biotech Ltd., Shanghai, China). The luminescence of firefly luciferase was determined using the Dual Luciferase Assay (Promega) following the manufacturer’s instructions.

### Statistics

Data were obtained from at least three replicates and expressed as mean ± standard deviation (SD). Student’s *t*-test and one-way analysis of variance (ANOVA) with Tukey’s adjustments were used for statistical comparison, with *P* < 0.05 as a level of statistical significance. Data entry, management, and analysis were conducted using SPSS 19.0 software (IBM, Armonk, NY, USA).

## Supplementary information

Author Contribution Form

## Data Availability

All data generated or analyzed during this study are included in this article. The data sets used and/or analyzed during the current study are available from the corresponding author on reasonable request.
