# Supplementary figures and images for: LncRNA SND1-IT1 facilitates TGF-β1-induced epithelial-to-mesenchymal transition via miR-124/COL4A1 axis in gastric cancer
Source: Cell Death Discov. 2022 Feb 19;8:73. doi: 10.1038/s41420-021-00793-6 (PMC8858320; doi:10.1038/s41420-021-00793-6)

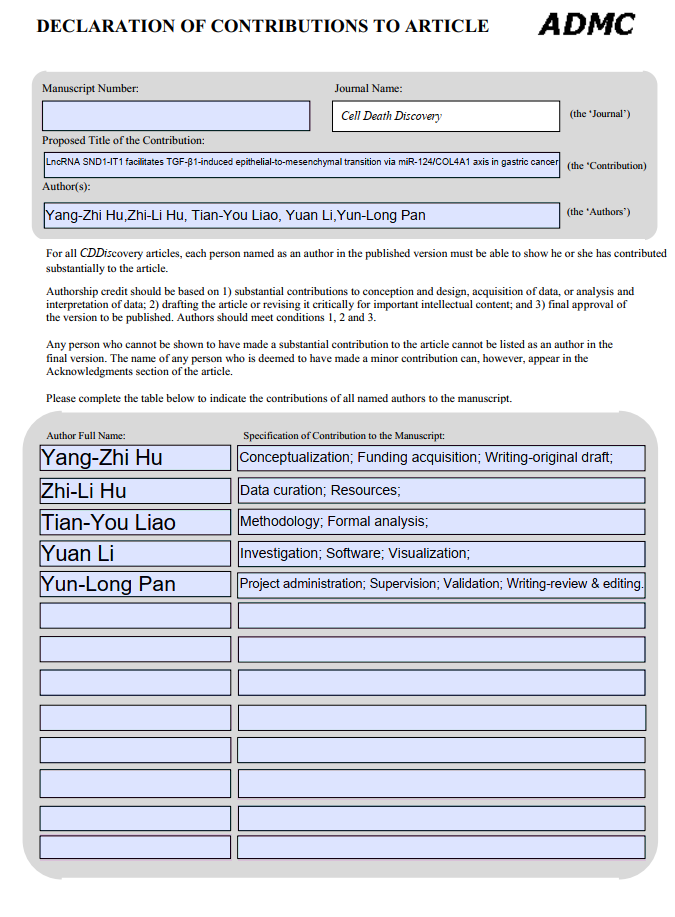

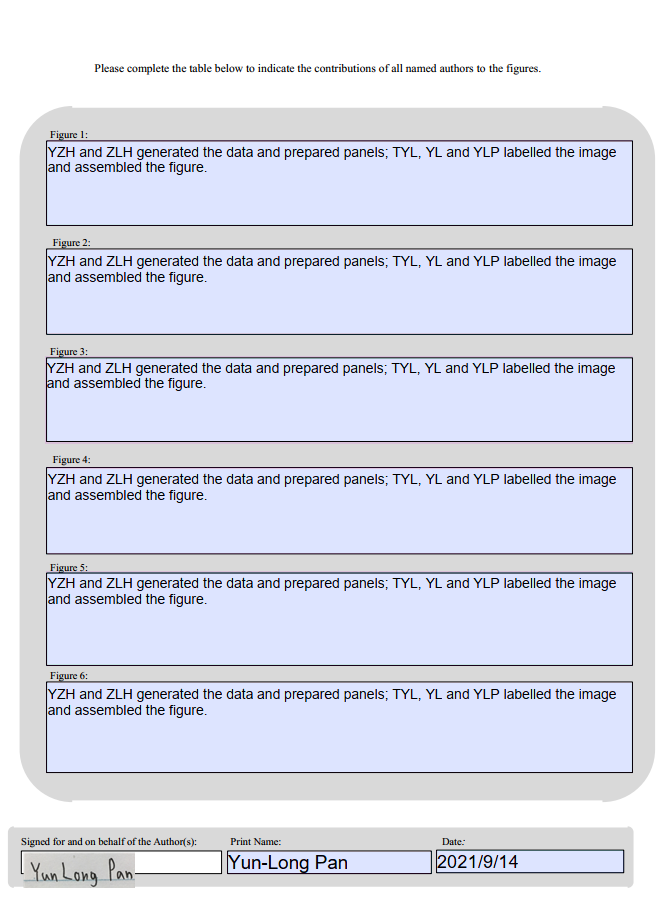

Supplement: Supplementary file 1 — Author Contribution Form [file 41420_2021_793_MOESM1_ESM.docx]
